# Supplementary material for: Clinicians’ and Users’ Views and Experiences of a Tele-Mental Health Service Implemented Alongside the Public Mental Health System during the COVID-19 Pandemic
Source: Int J Environ Res Public Health. 2023 May 19;20(10):5870. doi: 10.3390/ijerph20105870 (PMC10218653; doi:10.3390/ijerph20105870)
Supplement: Supplementary file 1 [file ijerph-20-05870-s001.zip › ijerph-2328071-supplementary.pdf]

Box S1. Survey questions

1. What were your initial impressions of telehealth to help people with mental health problems in Gippsland?
2. In Gippsland during the last 12 months was the use of telehealth encouraged with mental health clients?
3. How or what encouraged the use of telehealth?
4. If you are a mental health practitioner, do you use telehealth in your practice?
5. How do you use telehealth in your practice?
6. Please list the three most important things that enable your involvement in telehealth
7. Please list the three most important things that impede your involvement in telehealth
8. Could you tell us more about what does and does not work well in telehealth for persons with mental health problems?
9. Do you believe that telehealth could become an important part of how mental health services are provided?
10. What will need to change or be developed for telehealth to become part of routine care?

Table S1: Categories, codes and representative quotes for service provider and service user views on the use of telehealth as part of the HeadtoHealth program in Gippsland, Victoria

**Category**	**Code**	**Representative quote**
Conditions where use of telehealth is appropriate	In geographically isolated areas	Gippsland is a very large region and telehealth provides the option to connect and support with many people outside the immediate post code/without transport, etc.- *Mental health nurse*
	During Lockdown	A very helpful resource during lockdowns when people we forced to stay at home and couldn't travel. - *GP*<br>I don't have to drive anywhere to go and to see someone and especially in COVID times… you know, you're not out and about. So it's more convenient. And yeah, you can just sit at home and have a coffee or something if you want and it doesn't sort of encroach too much into your day. – *Service user 1 (F,47)*
	For technologically literate clients who have internet	Telehealth does make it very accessible and handy for someone like myself who's okay with the technology and has internet. - *Service user 17 (M, 45, LGBTQIA+)*
	Where there is an unmet need	Majority of clients were grateful to receive any support regardless of whether it is face to face or telehealth. – *Psychologist*<br>I had mental breakdowns while I didn't have an appointment or anything like that. And I had an email address one night, and I had sent an email through and I got an email right back saying, you know, 'These are the numbers you need to call if you feel stressed', you know, 'triple zero, or Lifeline or', you know, that kind of thing, which I had never had before in any other [service]. And that is a big thing. – *Service user 3 (M,28, Aboriginal)*<br>By the end of this phone call with someone I've never met before, I was smiling. I thought, 'I have help. I have somewhere to go. There's someone that gives a damn'. That's how I sort of summed it up at the end of the phone call. – *Service user 8 (F,64, Aboriginal)*
	Where services can stay connected to clients	Telehealth has ensured services have stayed connected with clients. It will be useful in the future used in a hybrid model with some meetings / appointments in person then follow up with telehealth. – *Social worker*
	Suitable age groups	Telehealth appears to be highly effective for clients aged approximately 15-55 as they are able to independently set up the telehealth call and open up via phone or video call. – *Psychologist*

	Organisational buy-in	Organisations had developed COVID working groups to support the implementation of service delivery through telehealth and in an online capacity. – *Families and housing worker*<br>Supportive schools allowing students to have telehealth sessions while at school. - *Psychologist*
	Helpful for clients with anxiety	Not ideal but extremely helpful especially with clients with anxiety etc – *Mental health support worker*
	Clients in recovery	It is helpful when the person is well and working on recovery maintenance. - *Mental health support worker*
	Where a therapeutic relationship already exists	It can work well especially in situations where a therapeutic relationship already exists. – *Social worker*
Conditions where telehealth may not be useful	Low SES groups	Some with MH issues can't afford a phone or phone credit - *Mental health support worker*
	Local stigma	Local stigma about Telehealth being a subpar service – *Mental health nurse*
	Clients at risk of suicide	It is difficult to have telehealth sessions with clients who are at risk of suicide as you have less control and knowledge of their surroundings. - *Psychologist*<br>[Not helpful for] those experiencing distress who need immediate connection with a human in person. – *Social worker*
	Culturally and linguistically diverse communities	I don't think telehealth is for everyone and those facing disadvantage - language and cultural barriers will do better with face to face. - *Social worker*<br>When I heard that I have to be on the phone. I was scared! Absolutely scared! And I thought, 'How will we make the connection by phone, when I know that my accent, my limitation and vocabulary and things like this plus the situation… a personal situation that is hard to open yourself when you don't know the other person?'. And um, well, [the clinician] showed me that … when you got [a] good professional, the other side who is trying to, you know … make you feel confident and you know, and trust in the other person and then and trust in the in the method that they're using and at the end I thought, 'Oh, [she] was lovely!'. – *Service user 2 (F,53, Culturally and Linguistically Diverse)*
	Children and older clients	Clients under the age of 15 require hands on therapy activities to be engaged and open up. Clients over the age of 55 tend have

		difficulty with technology and struggle to open up via telehealth. - *Psychologist*
Advantages of telehealth	Improves accessibility	Makes accessibility so much easier for patients. Also helpful to referring clinicians to be able to sit in for parts, ask questions and receive direct feedback. - *GP*
		Ease of service being delivered in comfort of own home, easer accessible and did not have to travel to Melbourne to see a specific mental health professional - *Psychosocial support worker*
		…I guess that having the telehealth was one of the reasons why I was able to see the psychologist almost straightaway. So that was a big bonus.- *Service User 7 (F, 23)*
		…being regional, you know, I wasn't going to travel to Melbourne; so it meant I had access to staff – *Service user 5 (F,39)*
	Provides privacy,	I specifically wanted telehealth because I didn't want to run into anyone I knew. Because I knew the worker who was doing the face to face services… and mostly for privacy it worked for me – *Service user 5 (F,39)*
	Phone can be used	Phoning clients means they are more likely to engage (even if they forget the appointment they are still happy to talk) - *Manager*
		I don't cope when there is too much stuff going on. And so those phone calls are just a godsend, because they call me I don't have to think about it. And sometimes it catches me out and like, sometimes I'll be driving and I'm just like I'm free to talk but I am driving [and] it will be crackly or whatever. Or you know, like sometimes (baby) is sleeping on me and I wake up and I'm like, 'Oh, hello!'. But, yeah, if I didn't get the phone call, I wouldn't get the appointment. – *Service user 6 (F,32)*
		I can build rapport and stuff with the psychologist perfectly fine over phone. I'm not a big fan of zoom sessions, I just find it really awkward. – *Service user 5 (F,39)*
		Clients don't have access to data and devices so phone calls are the primary mechanism of telehealth. - *Manager*
	When staff cannot work in the office	Staff being unavailable to work in the office so telehealth was required for them to work from home at certain times when needed. - *Mental health support worker*
		[Video conferencing] may sometimes provide a window into the person's environment. - *GP*

	Advantages of Video Conferencing	It would be helpful for clinicians to see the client's location as this would also allow clinicians to know the whereabouts of the client in case emergency services need to be dispatched. - *Psychologist*
Challenges in using telehealth	Not enough information on how to use telehealth	Feedback from participants was that even though it was helpful to not have to travel, not enough information was sent prior to the sessions and many didn't work as a result. - *Mental health support worker*<br>It helped people access services however, most required assistance from services to access. – *Mental health nurse*<br>[Practitioners used] poor technique in building relationships and rapport over phone or VC – *Social worker*<br>Telehealth did take both clinicians and clients time to adjust due to lack of face to face interactions. - *Psychologist*
	Less effective for building rapport	Less effective for establishing rapport and engagement - *Manager*<br>It can be harder to build a rapport over only phone or video interactions. - *Manager*<br>Very difficult to resource share and pick up body language / social cues. – *Social worker*
	Disadvantages of using telephone	Mental health nursing involves a lot of observation and engagement - not always able to be achieved as well over the phone. – *Mental health nurse*<br>Clients do not open up completely at times about their concerns resulting in missing information and short sessions. - *Mental health support worker*<br>Very impersonal- difficult to gage safety levels and mental state examinations over the phone. - *Psychosocial support worker*<br>Difficult if a client at risk doesn't answer. - *Mental health support worker*
	Video Conferencing not preferred	Patient preference is not for video in the youth population. - *GP*<br>VC not that accessible to low income clients. – *Manager*
	Lack of privacy in the home	People cannot often find a suitable location to have private discussions over video or phone. - *Administrator*
	Lack of internet, issues with technology	Clients in these far-reaching communities were relying on telephone service due to the [inadequate] internet capacities. - *Families and housing worker*

	communication and working devices	A lot of the time the appointments didn't happen, because you didn't have the zoom link and no one was there at reception to answer the calls or they were so busy you didn't hear from them for a few days. ... so it was just a lot of communication breakdowns, and a lot of missed appointments because of it. – *Service user 17 (M, 45, LGBTQIA+)*<br>When there is lagging or flat batteries etc it is very disruptive. – *Social worker*<br>I like the phone calls, because I can't think I can forget them…. I think the one time I missed a phone call…was … because my phone was flat. – *Service user 6 (F,32)*
	Impersonal	Many felt it was a poor substitute, they were not important as they were not sitting in a waiting room or with the professional. - *Mental health support worker*<br>It is not the same as face to face and can feel more distant and disconnected. – *Social worker*<br>Many clients find it uncomfortable and prefer face to face. - *Mental health support worker*<br>I would have preferred face to face and particularly in that sort of situation where the psychologist was making an assessment, I guess, where I was at. I think [one] gets a better sense of it if it was face to face…- *Service user 7 (F, 23)*<br>I get distracted very easily, especially over the phone. Speaking to people over the phone, all I'm looking at is a blank screen on my phone, or I'm just looking at my hands. When I have my moments of shutting down, it's very easy to think, 'Oh, maybe she's just thinking'. Whereas in person, at least I hope it is more obvious that I'm [not] feeling very comfortable with what I'm speaking about. And I feel that in person, people give me a little bit more leeway to help improve my comfort level. Whereas on the phone, I am very much on my own. People can't see my reactions. – *Service user 14 (F, 37)*
Client outcomes with telehealth	When client prefers telehealth	[It's] dependent on the preference of the client… There are some families and clients who are benefiting dramatically from the use of a Telehealth system- maintaining set appointments and also engaging in meaningful therapeutic level counselling with service providers. - *Families and housing worker*

	Lack of face to face interactions caused distress for Clients in isolation	Clients felt the impact of not having face to face, describing significant distress from isolation. - *Manager*<br><br>Participant's symptoms being exacerbated by screen/phone interaction. - *Mental health support worker*
	May discourage clients from accessing services	I am concerned it could further exacerbate some people [not] accessing the service as it can be isolating. People not leaving their home with no real social connections. - *Family violence service worker*
	Unhelpful for housebound clients	Does not challenge people who have difficulty leaving their homes. – *Social worker*
Recommendations for future use	Best used in existing relationship	Things work best when there is an existing relationship. – *Social worker*
	Better as part of dual mode of delivery	Methods to address the need for clients to somewhat have a personal connection. May require blended delivery. - *Manager*<br>It is not a most preferred option as a sole delivery of service, but will be accommodated if it is part of a dual mode of service delivery. - *Mental health support worker*
	Prior communication with client on how to use technology	Clear communication with clients on methodology for appointments. – *Social worker*
	Screening clients before use	I believe, if not already occurring that there is a screening tool for being 'Telehealth Ready'. If the client does not interact socially, I don't believe this way of support is beneficial. - *Family violence service worker*
	Technology needs to improve	It's still early days, the technology needs to grow and be easy to access and trusted by clients. - *Occupational therapist*<br>Access to an appropriate privacy medium so [that there is no need to]… give patient personal number/email. - *GP*
	Training practitioners	Practitioners receiving some training in the technology and ways to promote connectedness. – *Social worker*
	Establishing telehealth kiosks	It would be beneficial for there to be accessible [kiosks] were people can travel and access computers to engage in telehealth sessions. This would ensure that they have access to technology,

	reception, internet and ensure they are in a safe private environment where they can open up. - *Psychologist*
